# The four-factor personality model and its qualitative correlates among opioid agonist therapy clients

**DOI:** 10.3389/fpsyt.2023.1129274

**Published:** 2023-06-09

**Authors:** Ioan T. Mahu, Patricia J. Conrod, Sean P. Barrett, Aïssata Sako, Jennifer Swansburg, Sherry H. Stewart

**Affiliations:** ^1^Department of Psychology and Neuroscience, Life Sciences Centre, Dalhousie University, Halifax, NS, Canada; ^2^Ste-Justine Hospital, Centre de Recherche, Montreal, QC, Canada; ^3^Department of Psychiatry and Addictology, University of Montreal, Montreal, QC, Canada; ^4^Department of Psychiatry, QEII Health Sciences Centre, Dalhousie University, Halifax, NS, Canada; ^5^Quebec-Atlantic Node, Canadian Research Initiative in Substance Misuse, Centre de Recherche du CHUM, Montreal, QC, Canada

**Keywords:** qualitative–quantitative analysis, personality, opioid agonist therapy, methadone, polysubstance use, motives

## Abstract

**Background:**

The Four Factor Personality Vulnerability model identifies four specific personality traits (e.g., sensation seeking [SS], impulsivity [IMP], anxiety sensitivity [AS], and hopelessness [HOP]) as implicated in substance use behaviors, motives for substance use, and co-occurring psychiatric conditions. Although the relationship between these traits and polysubstance use in opioid agonist therapy (OAT) clients has been investigated quantitatively, no study has examined the qualitative expression of each trait using clients’ voice.

**Method:**

Nineteen Methadone Maintenance Therapy (MMT) clients (68.4% male, 84.2% white, mean age[SD] = 42.71 [10.18]) scoring high on one of the four personality traits measured by the Substance Use Risk Profile Scale [SURPS] completed a semi-structured qualitative interview designed to explore their lived experience of their respective trait. Thematic analysis was used to derive themes, which were further quantified using content analysis.

**Results:**

Themes emerging from interviews reflected (1) internalizing and externalizing symptoms, (2) adversity experiences, and (3) polysubstance use. Internalizing symptoms subthemes included symptoms of anxiety, fear, stress, depression, and avoidance coping. Externalizing subthemes included anger, disinhibited cognitions, and anti-social and risk-taking behaviors. Adverse experiences subthemes included poor health, poverty, homelessness, unemployment, trauma, and conflict. Finally, polysubstance use subthemes include substance types, methods of use, and motives. Differences emerged between personality profiles in the relative endorsement of various subthemes, including those pertaining to polysubstance use, that were largely as theoretically expected.

**Conclusion:**

Personality is associated with unique cognitive, affective, and behavioral lived experiences, suggesting that personality may be a novel intervention target in adjunctive psychosocial treatment for those undergoing OAT.

## 1. Introduction

Now in its fourth wave, the opioid epidemic continues to cause havoc and destruction for individuals, families, and communities (1). Opioid agonist treatment (OAT), such as methadone or buprenorphine/naloxone, has been extensively shown to be effective at reducing opioid-related harms (2, 3). Nonetheless, the majority of persons who use opioids engage in polysubstance use (4), contributing to a more complicated clinical profile that is not always fully addressed by OAT alone. Indeed, the clinical profile typical of people who use opioids includes not only polysubstance use (5), but also high rates of trauma exposure, poverty, criminal justice system involvement (6–8), comorbid psychopathology (9), and other comorbid health problems such as chronic pain (6–8). In addition, polysubstance use is also associated with poorer treatment outcomes (10), including decreased retention rates (11), additional health-related complications (12), and higher mortality rate (13).

Given this increased clinical complexity, and to enhance the effectiveness of OAT, psychosocial interventions are recommended as a crucial component of treatment across several OAT clinical guidelines (14–17). A systematic review by Dugosh et al. (18) largely supports the use of psychosocial interventions in the context of OAT, although the added benefit does tend to vary across medications (methadone, buprenorphine/naloxone), outcomes (e.g., illicit opioid use, treatment adherence, HIV risk, psychosocial functioning, and adherence to psychiatric medication), and psychosocial intervention types. One such model could include personality-targeted interventions (19). These can be brief while maintaining high levels of efficacy due to their targeted nature, and impact multiple outcomes, including polysubstance use and mental health (19, 20). These interventions have been used with great success among youth as a brief model for reducing both substance use and mental health problems (20) but have not yet been adapted to the Methadone Maintenance Therapy (MMT) setting.

Personality-targeted interventions are based on the four-factor personality risk model (21–23), which outlines how four lower-order traits are differentially associated with substance use vulnerability through specific neurological and motivational mechanisms. These four traits include: (1) sensation-seeking (SS), defined as the preference for novel and exciting stimuli, (2) impulsivity (IMP), defined as deficits in behavioral inhibition and planning, (3) hopelessness (HOP), operationalized as depression proneness and pessimism about the future, and (4) anxiety-sensitivity (AS), defined as the fear of one’s bodily arousal sensations. Each trait is associated with preference for specific substances, motives for use (i.e., reasons for using drugs, including but not limited to: enhancement, social, conformity, and coping motives), and co-occurring psychiatric conditions [for a review, see (21)].

Recent work by our group has explored the impact of these high-risk personality traits on substance use (24) and substance use motives (25) among MMT clients, providing emerging evidence that personality may be a suitable target for focused intervention. For example, SS was associated with past 30-day use of alcohol, cannabis, and stimulants (24). IMP was associated with past 30-day injection drug use (24). HOP was associated with past 30-day opioid and tranquilizer use (24). Finally, AS was associated with past 30-day tranquilizer use (24).

Although we have some evidence that personality is implicated in the maintenance of addictive behavior among MMT clients (24), adapting existing personality-targeted interventions to the MMT setting requires a more nuanced understanding of the ways in which personality relates to polysubstance use in this population. This is necessary not only to adapt the theoretical underpinnings of the four-factor model to this new population, but also to design appropriate intervention materials (e.g., treatment manuals) and identify potential intervention outcomes beyond reducing substance use. Consequently, this study was designed to address this gap using qualitative methodology, aimed at gaining a better understanding of how personality traits from the four-factor model are expressed in relation to high-risk behaviors including polysubstance use among MMT clients. Qualitative methodologies allow for the extraction of “themes” from coded interview data. We were specifically interested in learning more about how each of the four high-risk personality traits is expressed cognitively, affectively, and behaviorally in relation to various lived experiences and how these experiences relate to substance use motives and behaviors among a sample of high personality risk MMT clients. We were also interested in the relative endorsement of each theme within each specific personality profile.

## 2. Materials and methods

### 2.1. Participants

Twenty OAT clients who took part in a previous quantitative study (24, 25), recruited from one of four OAT clinics in Montreal (*n* = 2) or the Halifax Regional Municipality (*n* = 2), were invited to take part in an in-depth semi-structured qualitative interview. At the time that recruitment took place, the predominant form of OAT at our participating clinics was methadone, and as such we restricted participation to MMT clients to control for any potential differences that may emerge between different OAT types (e.g., more flexible take-home dosages with buprenorphine/naloxone). Participant demographics are reported in Table 1. Clients scoring at least one standard deviation or higher relative to the validation sample reported by Woicik et al. (26) on either one of the four personality profiles measured with the Substance Use Risk Profile Scale [SURPS; (26)] were invited to participate, until five individuals were recruited for each personality profile. If participants met this criterion on more than one trait (which occurred in 73.8% of cases), we prioritized recruiting them for an interview in their highest relative elevation first, unless the recruitment target of five was already met for that subgroup. We recruited 5 HOP clients, 5 SS clients, 4 AS clients, and 6 IMP clients.^1^ One of the HOP interviews was removed from the analysis after detecting that the interviewed participant did not meet the required elevation in the HOP trait due to a data entry error prior to recruitment, leading to a final sample of 19 participants (4 HOP, 5 SS, 4 AS, and 6 IMP). Personality characteristics in the overall sample and each individual personality interview are reported in Table 2.

**Table 1:** Participant demographics.

Variable	Counts	% of Total	Cumulative %
*Gender*			
Men	13	68.4%	68.4%
Women	6	31.6	100.0%
*Employment*			
Unemployed	6	33.3%	33.3%
Social assistance	1	5.6%	38.9%
Employed	8	44.4%	83.3%
Disabled	3	16.7%	100.0%*
*Ethnicity*			
Indigenous/Aboriginal/First Nations	2	10.5%	10.5%
Black, Afro-Canadian, Caribbean-Canadian	1	5.3%	15.8%
White	16	84.2%	100.0%
*Highest education completed*			
Elementary school	2	10.5%	10.5%
Junior High school	3	15.8%	26.3%
High school	8	42.1%	68.4%
Trade school	3	15.8%	84.2%
Community School	1	5.3%	89.5%
Some university/college	1	5.3%	94.7%
University/college degree	1	5.3%	100.0%
*Relationship status*			
Single (never married)	10	52.6%	52.6%
Married/Cohabitating	3	15.8%	68.4%
Separated/Divorced	5	26.3%	94.7%
Common Law	1	5.3%	100.0%
*Current living arrangements*			
Renting	12	63.2%	63.2%
Own your own home	1	5.3%	68.4%
Living with family (not paying rent)	2	10.5%	78.9%
Community shelter/ transitional housing	2	10.5%	89.5%
Living with a roommate	2	10.5%	100.0%
*Yearly income*			
$0 to $10,000	7	36.8%	36.8%
$10,001–$20,000	7	36.8%	73.7%
$20,001–$29,000	2	10.5%	84.2%
$59,001–$60,000	1	5.3%	89.5%
$79,001+	1	5.3%	94.7%
Other	1	5.3%	100.0%
*Other demographics*	Counts	Mean	SD
Age (years)	17*	42.71	10.18
Daily Methadone Dose (mg)	19	85.32	39.83

*Two participants did not provide data on age. One participant did not provide data on employment.

**Table 2:** SURPS personality scores by interview group.

Interview group	*N*	Personality trait	Mean	SD	Mean *Z*-score
Sensation-seeking	5	**SS**	**21.2**	**2.387**	**1.90**
		IMP	11.6	2.966	0.19
		AS	12.2	3.701	0.00
		HOP	15.4	3.782	0.42
Impulsivity	6	SS	14.8	3.710	−0.22
		**IMP**	**16.3**	**1.366**	**1.94**
		AS	15.3	3.077	1.12
		HOP	17.0	4.775	0.81
Anxiety-sensitivity	4	SS	16.3	3.096	0.25
		IMP	12.8	1.366	0.61
		**AS**	**16.8**	**1.258**	**1.63**
		HOP	13.3	1.826	−0.11
Hopelessness	4	SS	13.0	1.414	0.83
		IMP	13.5	0.577	0.89
		AS	13.0	1.414	0.46
		**HOP**	**20.0**	**1.826**	**1.54**
Overall sample	19	SS	17.47	3.66	0.66
		IMP	13.74	2.66	0.98
		AS	14.32	3.09	0.76
		HOP	16.42	4.17	0.66

Personality traits (*n* = 19)	>1 SD Counts (%)
Sensation-seeking	6 (31.6%)
Impulsivity	12 (63.2%)
Anxiety-sensitivity	10 (52.6%)
Hopelessness	7 (36.8%)

SS, sensation-seeking; IMP, impulsivity; AS, anxiety-sensitivity; HOP, hopelessness; SD, standard deviation; SURPS, substance use risk profile scale; Mean *Z*-Score reflects the standardized personality score, derived from adult norms reported by Woicik et al. (40).Bold values serve to visually highlight the SURPS score of the relevant personality trait within different interview groups.

### 2.2. Data collection

Semi-structured interviews lasted 60–90 min and were conducted in a private room at each OAT clinic by experienced interviewers (two doctoral candidates in clinical psychology, and 1 senior research assistant). Interviews were conducted in either English (*n* = 17) or French (*n* = 3), as per participants’ preference. Informed consent was obtained at the outset of the session, and participation was voluntary. Participants were informed that participation in the study would not affect their OAT treatment, and that details from the interview would remain confidential. They were informed that their composite experiences would be used in the creation of scenarios for an upcoming intervention and that we would take care to protect their confidentiality (e.g., not using identifying information, combining stories across different people, etc.) when using their experiences to create scenarios. Interviews were audio recorded for transcription and analysis purposes. Identifying information was removed from the final transcripts. Participants were compensated with $20 CDN at the end of the interview. Ethical approval to conduct this research was obtained *via* each relevant hospital research ethics board in Montreal and the Halifax Regional Municipality.

The semi-structured interview guide was designed to gather information to support the eventual development of scenarios and material for a future personality targeted intervention with OAT clients. This was similar to the strategy used for developing other adaptations of the personality-targeted intervention [e.g., (27, 28)]. Briefly, we collected information regarding treatment goals, barriers, needs, and personality-specific information regarding the cognitive, affective, and behavioral lived experience of the personality trait that was elevated in the individual participant. More specifically, participants were invited to describe past situations where their personality led them to experience an unfavorable outcome. Substance use and substance use motives were specifically queried if they did not emerge organically in the scenarios described. Open ended questions and more specific probes were used as necessary to obtain sufficient detail (see Supplementary materials for a copy of the interview guide).

### 2.3. Data analysis

Completed interviews were transcribed verbatim into either English or French. The first author, who is bilingual, listened to each interview to double check that the transcriptions were accurate. Data was imported for management, coding, and analysis into NVivo Pro v. 12 (QSR International, 2021), a qualitative data analysis software package. Thematic analysis (29) was used to derive codes and interpret the final themes that emerged from the data. To aid in the description and comparison of themes across personality profiles, we also employed content analysis (30) as a secondary analytic strategy, as it allowed us to quantify and compare the endorsement of themes across different personality groups by counting the number of references (i.e., coded units) belonging to each theme. We then examined the relative endorsement of each theme as a percentage of total references within each personality group.

The epistemological position employed during analysis was realist/essentialist, and focused on reporting the experiences, meaning, and reality of participants. Initial codes were derived by the first author (ITM), who at the time was a senior doctoral candidate in clinical psychology. ITM has 9 years of research experience with the four-factor personality model in the context of addiction and polysubstance use, knowledge of the OAT population, and over 6 years of supervised clinical training (>1,500 h) in cognitive-behavioral assessment and therapy. Coding focused primarily on semantic content (i.e., explicit, surface meaning in text), unless the data provided strong contextual evidence of an implicit meaning. Additionally, we used a combination of both deductive and inductive coding strategies. For example, initial codes were deductive, and informed by the structure of the interview guide and the framework of the cognitive behavioral model (i.e., separating thoughts, behaviors, and affect, within the context of triggering situations). However, other codes emerged inductively through familiarization with the data. Codes and themes were collaboratively revised among the authors until consensus was reached. Following a variety of revisions, final codes were organized into themes (*n* = 28; collection of thematically similar codes) and theme families (*n* = 3, collection of similar themes) based on the initial research question and the authors’ combined clinical and research experience with personality, addiction, and psychopathology.

## 3. Results

Three primary theme families emerged from the interviews: (1) internalizing and externalizing symptoms, (2) adversity experiences, and (3) polysubstance use.

### 3.1. Internalizing and externalizing symptoms

One of the major themes that emerged from participants’ stories about their personality reflected symptoms of internalizing (i.e., anxiety, depression, avoidance) and externalizing (i.e., anger, disinhibition, thrill seeking, anti-social behavior) forms of psychopathology. These various subthemes were expressed through coding of various affective (i.e., how they felt emotionally or physically), cognitive (i.e., how they interpreted events and how they thought in different situations), and behavioral (i.e., how they acted) processes that participants described in various situations. The internalizing symptoms included experiences of (1) anxiety, fear, or panic, (2) depression or low mood, and (3) avoidant coping behaviors. The externalizing symptoms included experiences of (4) anger or frustration, (5) disinhibited cognitions, (6) anti-social behaviors, and (7) thrill-seeking or risk-taking. Table 3 depicts the distribution of total references coded for these themes within each personality profile. Each is described in more detail below.

**Table 3:** Internalizing and externalizing symptoms by personality group.

Category	Themes	SS (5)	IMP (6)	AS (4)	HOP (4)	Total (19)
Externalizing symptoms	Anger or frustration	1.79%	9.12%	1.2%	7.45%	6.32%
	Disinhibited cognitions	3.57%	21.28%	4.82%	2.13%	12.48%
	Thrill seeking and risk taking	41.96%	9.12%	2.41%	6.38%	14.02%
	Anti-social behaviors	18.75%	19.59%	2.41%	13.83%	16.07%
Internalizing symptoms	Anxiety, fear, or panic	8.04%	16.22%	55.42%	12.77%	19.66%
	Depression or low mood	23.21%	19.93%	13.25%	47.87%	24.1%
	Avoidant coping behavior	2.68%	4.73%	20.48%	9.57%	7.35%
	Total % (# references coded)	100% (n = 112)	100% (n = 296)	100% (n = 83)	100% (n = 94)	100% (n = 585)

SS, sensation-seeking; IMP, impulsivity; AS, anxiety-sensitivity; HOP, hopelessness. Number in parentheses indicates the number of interviews conducted with each profile. Cells are color heat mapped to visually indicate the relative endorsement of each theme within each respective personality group. Red indicates high endorsement, yellow/orange is moderate endorsement, and green is low endorsement.

#### 3.1.1. Anxiety, fear, or panic

A common affective experience was general feelings of anxiety, which included discussions around feeling anxious, afraid, or stressed, experiencing panic attacks or panic symptoms, and catastrophizing about physical sensations. These descriptions often highlighted symptoms of a panic attack. For example, one participant described his panic attacks as follows:

“When I start having that, and then I start, *sighs* having like, breathing starts getting like caved in and I'm like oh fuck it, and I start getting palms sweaty, feet tingling, hands start tingling and then it [heart-rate] starts going going going going going going going.” (C113, male, age 33, AS)

Unsurprisingly, many participants described being stressed because of the multiple barriers and hardships they needed to navigate daily. These included experiencing poverty, homelessness, marginalization, and interpersonal conflict or violence. These feelings of stress were closely related to substance use, often leading to substance use as a form of coping, as explained by one participant: “the main reason I smoke marijuana now too …at-you know three, four grams a day is a lot for just one person … and the only reason I’m using so much right now is ‘cause I’m all stressed out.” (A105, male, age 48, IMP).

Relative to other personality traits, descriptions of this kind were found to dominate the content of discussions within the AS interviews, occupying 55.42% of total internal experience references coded (Table 3). Closely connected to the theme of fear and stress were descriptions of symptoms of withdrawal states, which were frequently discussed among high AS MMT clients specifically.

#### 3.1.2. Depression or low mood

Feelings of intense sadness, depression, loneliness, negative self-talk, regrets, guilt, and suicidal or self-injurious behavior were combined to reflect an internal experience of sadness or depression. For some, these feelings were connected to situations in which they experienced a great loss or some other major life stressor, and were often directly followed by substance use or polysubstance use as a means of coping:

“Lost, lost the kids. She didn't want me to know where they were. Who had them, and, so yeah… Uhm, took a toll on me. I, I lost it. I didn't want to answer the door to nobody. I didn't want no one around me. And, uh, my daughter's mom, she had gotten an apartment in town and moved out of my house and of course I locked myself in my home. For, thirty some days and… Drink, started doing drugs, and um, led to other drugs.” (A154, male, age 43, IMP)

For others, this theme was evident in descriptions containing negative self-talk or rumination.

“Yeah, and-and that's where I get depressed some-sometimes because I did it to myself, right? Nobody did it to me, nobody made me do any of the things that I did so that plays a big thing in the back of your head, like you’re an idiot, why'd you even do that? Look what you had, what you don't have, look at what happened, this and that and um you know, you still choose to do it again.” (A118, male, age 42, HOP)

Some participants expressed regret or remorse about past behavior or events, particularly when the consequences were severe and resulted in loss of employment, relationships, and/or opportunities. Participants described making bad decisions in the moment, without considering the potential consequences, but with time realizing the cost of their actions and experiencing regret over “what could have been.”

Relative to other personality groups, this theme was particularly prominent among the internal experiences of HOP interviews, occupying 47.87% of total references coded in this group (Table 3).

#### 3.1.3. Avoidant coping behaviors

Avoidance, distraction, safety-behaviors, thought suppression, and interpersonal withdrawal as means of coping with heightened anxiety or other distressing emotional states comprised a subtheme indexing a variety of avoidant coping behaviors.

“It's just reading, it's just like taking my focus away from everything around me or whatever is, is making me anxious and just kind of like um, just kinda like having something to like focus on. Like I'll even just like don't matter what's in my purse if there's anything to read, even if it's like a grocery list, I can just read it like for that few minutes just to make my head go back, to like make my mind go back.” (A202, female, age 26, AS)

These strategies were frequently discussed by those in the AS group (20.48%), particularly for coping with anxiety and panic attacks (Table 3).

#### 3.1.4. Anger or frustration

Anger was an emotion often endorsed in relation to dealing with conflict, loss, poverty, or marginalization. For example, a participant described his frustration with accessing pain medications for a painful surgery when the system labeled him as an “addict”, explaining:

“We [doctors] don't prescribe them to people like you anymore. *laughs* To give them to me for years and then they tell me they can't, I can't have anymore? That's when I get in trouble, that's when I have a problem, and that is why I have such a deep-seated hate for the medical society and stuff … just because of stuff they've done. You don't always get treated well in the healthcare system if you’re an addict. You know, everybody else says, it's a problem because you are a problem and you're costing money to taxpayers and you're this and you're that, they don't let you forget those things easy either. So, between what doctors kinda put me through and jerk me around you know I just don't like doctors and I don't like healthcare facilities and places like that very well anymore because I just don't trust them, you know.” (A105, male, age 48, IMP)

Other participants described affective reactions of anger linked with an overall difficulty in regulating their own emotions or linked with a tendency to jump to conclusions. This sub-theme was more commonly endorsed among the high IMP interviews (9.12%) relative to the other personality traits (Table 3).

#### 3.1.5. Disinhibited cognitions

Codes reflecting a collection of cognitions (or impulses) that described a tendency to approach situations without thinking through consequences and feeling as if one has no control over their own actions, encompassed a theme of disinhibited or impulsive cognitions. Codes within this theme often co-occurred with other externalizing symptoms, such as anger/frustration and anti-social behavior.

“But… I mean I’ve got a very short fuse and I go from zero to five thousand in, you know, a very short time and …I’ve always been very impatient, very…you know …It’s just always been…it’s just been go, go, go, go, go and I seem to can’t slow down or relax or-or let myself relax, I feel like I got to be… right on edge all the time and that’s very tiring.” (A105, male, age 48, IMP)

Some participants described feeling as if they had little control over their own actions, and that their behaviors occurred quite automatically without much planning or forethought:

“Well, when it's happening, I don't ... it's not a plan or anything, it just, it just happens. It's after that I would, see any uh, any kind of plan, or anything, but not, not while. It's automatic. I don't know if that makes sense, but it's automatic.” (A103, male, 61, SS)

Relative to other personality groups, these disinhibited cognitions were commonly endorsed within the interviews with high IMP clients (21.28% of coded references; Table 3).

#### 3.1.6. Anti-social behaviors

Instances of aggression (e.g., getting into fights), committing criminal acts (e.g., stealing, armed robbery), or engaging in interpersonal deception (e.g., lying to conceal substance use) were coded under this theme. These behaviors were generally described as a desperate last-ditch attempt to acquire money or drugs, and often motivated by a desire to avoid painful and uncomfortable withdrawal sensations.

“I didn't have any money left, and I didn't have anything else left, and I had to have the drugs, I was sick. I did what I really didn't want to do [robbing a pharmacy], ‘cause I knew what was gonna happen from the first one [first incarceration].” (A105, male, age 48, IMP)

Experiencing poverty was heavily tied to coded anti-social behaviors, as were descriptions of disinhibited cognitions. This theme was frequently endorsed among SS (18.75%), IMP (19.59%), and HOP (13.83%) interviews (Table 4). However, the qualitative expression of this theme differed across these three traits. Relative to the other two traits, the anti-social behaviors described in HOP interviews primarily featured coded references to deception (e.g., lying to hide information from loved ones) largely to avoid inter-personal conflict. In contrast, those in IMP interviews featured more references to aggression (i.e., being involved in fights or violent actions) and criminal activity (e.g., robberies), while those in SS interviews primarily featured references to criminal activity.

**Table 4:** Adversity experiences by personality group.

Themes	SS (5)	IMP (6)	AS (4)	HOP (4)	Total (19)
Health	24.64%	28.28%	40.0%	34.88%	29.44%
Poverty, homelessness, and unemployment	14.49%	14.14%	30.0%	18.6%	16.45%
Trauma	52.17%	28.28%	0.0%	23.26%	32.03%
Interpersonal conflict	8.7%	29.29%	30.0%	23.26%	22.08%
Total % (# references coded)	100% (*n* = 69)	100% (*n* = 99)	100% (*n* = 20)	100% (*n* = 43)	100% (*n* = 231)

SS, sensation-seeking; IMP, impulsivity; AS, anxiety-sensitivity; HOP, hopelessness. Number in parentheses indicates the number of interviews conducted with each profile. Cells are color heat mapped to visually indicate the relative endorsement of each theme within each respective personality group. Red indicates high endorsement, yellow/orange is moderate endorsement, and green is low endorsement.

#### 3.1.7. Thill seeking and risk taking

This theme describes a way of interacting with the environment that is cognitively and affectively centered around chasing rewards and thrills, and behaviorally comprised of rule breaking or risk-taking behaviors.

“I uh, I always used to call it the gambler's rush. Your heart starts beating fast. your breath - your breathing starts getting heavy and that, right? Your blood pressure goes sky high.” (A103, male, 61, SS)

These behaviors often included descriptions of various high-risk (e.g., speeding, dangerous driving) and rule-breaking (e.g., cheating at cards, trespassing, vandalism) activities that contained an element of excitement and reward. This theme was heavily endorsed within the SS interviews (41.96% of external references coded; Table 3).

### 3.2. Adverse experiences

The next theme family reflected adverse experiences or situations that were frequently mentioned in various interviews. This theme was often intertwined with the previously discussed symptoms of internalizing and externalizing psychopathology, often as an antecedent or consequence. The major subthemes here were: (1) Health, (2) Poverty, Homelessness, and Unemployment, (3) Traumatic Experiences, and (4) Interpersonal Conflict. The endorsement of each of these subthemes by personality can be seen in Table 4. More detail on these subthemes appears next:

#### 3.2.1. Health

This theme included descriptions of poor physical health or chronic pain, either due to age, chance, or accidents. For some participants, managing pain was listed as a major contributor to developing an addiction in the first place: “I started getting back to work, I got back to work and then I broke my foot. Doctor [name] put me on a couple pain medications, the next thing you know, I’m hooked on pills *participant laughs*.” (A118, male, age 42, HOP). Relative to other adverse experiences, poor physical health was frequently discussed in HOP (34.88%) and AS (40%) interviews (Table 4).

#### 3.2.2. Poverty, homelessness, and unemployment

Some participants described experiencing homelessness, unemployment, job loss, and poverty. This contributed to experiencing high levels of stress, social isolation, and marginalization; these descriptions did not appear to differ much by personality traits (Table 4). Combined with an active addiction, and the need to avoid withdrawal states, some participants described circumstances of poverty pressuring them into crime as a way of financially supporting their substance use.

“Imagine if you need that to… and you have no money. You have to go find money first then go. And when you're going to look for money, you're so weak, that you can't do anything anyways, so … you're basically crawling on the streets looking for money.” (E130, male, age 46, IMP)

#### 3.2.3. Trauma

Many participants described various traumatic situations, ranging from childhood abuse, sexual violence, interpersonal violence, operational traumas, accidents, and death or near-death experiences (e.g., witnessing friends overdose or overdosing themselves). These scenarios were often described as an important contributor to developing and maintaining later substance use or anti-social behavior.

“But I used to use...just to escape...an' bein'- bein' sexually abused... Bein' beaten, by my dad an' stuff. And as I got older I- like I never dealt with it when I was younger. Then when I hit like eighteen, nineteen... I was out breakin' the law... goin' back and forth to jail... it just… one thing led behind- after another just my life spun out of control.” (B143, male, age 42, IMP)

Interestingly, trauma was frequently discussed in the SS interviews (51.43% of endorsed adversity references, Table 4); however, closer inspection reveals that these references were mostly concentrated in one SS interview with a participant that disclosed being diagnosed with post-traumatic stress disorder because of a job-related traumatic exposure. He explained being attracted to a career as a coast guard because of the excitement the job offered, but that he eventually turned to drinking and substance use as a means of coping with painful memories of traumatic rescue attempts. Surprisingly, trauma was not discussed at all in the AS groups (0% of coded references).

#### 3.2.4. Interpersonal conflict

This theme included codes centered around behaviors that describe various forms of interpersonal relationship ruptures. Participant experiences of feeling marginalized or stigmatized by society are also included here. This theme often overlapped with the “deception” component of the anti-social behavior external theme. Many participants described the major impact that addiction has had on their social network, including needing to hide their polysubstance use behaviors from others, and the devastating consequences to their interpersonal relationships when they could no longer keep up an act.

“I-you know what, I honestly don't know how I was able to hide it for so long. Like, without, you know, my wife knowing at all. It … still amazes me to this day how I could hide it for that long of a time. She got me though, she dug in my pockets one night I was sleeping *participant laughs*. She pulled it, “what the hell is this?” big old bag of, uh, I can't even remember how I responded. I don't think I said too much.” (A118, male, age 42, HOP)

Interpersonal conflict was moderately endorsed by all profiles, except for SS which had low endorsement of this theme (8.7% of references coded; Table 4).

### 3.3. Polysubstance use

Discussions around substance use featured heavily throughout all interviews, and mainly clustered around (1) substance type and (2) substance use motives (i.e., reasons for substance use). Participants also spoke about various methods of use (e.g., injection, snorting, oral) and maintaining factors for their use (e.g., craving, withdrawal symptoms, and social network use).

#### 3.3.1. Substance type

Consistent with polysubstance use, participants described using a wide range of substances, including alcohol, tobacco, cannabis, opioids, stimulants, tranquilizers, hallucinogens, and ecstasy. The relative distribution of coded references across each drug category among each personality group is shown in Table 5. Of note, IMP showed non-specificity with relatively equal endorsement across several drug categories: alcohol (13.85%), tranquilizers (19.49%), cannabis (21.54%), and opioids (24.62%). In contrast, SS interviews frequently referenced alcohol (25.34%) and opioids (34.93%), AS frequently referenced tranquilizers (35.21%) and opioids (21.13%), while HOP frequently referenced stimulants (35.82%), opioids (34.44%), and drugs non-specifically (20.9%; i.e., mentioned substance use but did not specify which type).

**Table 5:** Drug type by personality.

Drug type	SS (5)	IMP (6)	AS (4)	HOP (5)	Total (20)
Alcohol	25.34%	13.85%	4.23%	1.49%	14.2%
Benzos	2.74%	19.49%	35.21%	0%	13.99%
Cannabis	12.33%	21.54%	1.41%	4.48%	13.36%
Cigarettes	1.37%	1.03%	8.45%	1.49%	2.3%
Hallucinogens	5.48%	0.51%	2.82%	1.49%	2.51%
Opioids	34.93%	24.62%	21.13%	34.33%	28.6%
MDMA	0%	0%	5.63%	0%	0.84%
Stimulants	10.27%	6.15%	12.68%	35.82%	12.53%
Drugs (unspecified)	7.53%	12.82%	8.45%	20.9%	11.69%
Total % (# references coded)	100% (*n* = 146)	100% (*n* = 195)	100% (*n* = 71)	100% (*n* = 67)	100% (*n* = 479)

SS, sensation-seeking; IMP, impulsivity; AS, anxiety-sensitivity; HOP, hopelessness. Number in parentheses indicates the number of interviews conducted with each profile. Cells are color heat mapped to visually indicate the relative endorsement of each theme within each respective personality group. Red indicates high endorsement, yellow/orange is moderate endorsement, and green is low endorsement.

#### 3.3.2. Substance use motives

Motives for use included (1) conformity, (2) enhancement, (3) expansion, (4) social, (5) pain relief, (6) coping with anxiety, stress, or trauma, (7) coping with depression, and (8) coping with withdrawal. For brevity, example excerpts for each motive can be found in Table 6. Overall, all personality profiles referenced more frequent negative reinforcement motives (e.g., coping motives) relative to positive reinforcement (i.e., social, enhancement, or expansion motives). Notably, all referenced enhancement motives to some extent (lowest for AS at 20.69% and highest for SS at 29.69%). However, this relative preference for negative reinforcement was more pronounced for the internalizing personality traits (AS and HOP) relative to the externalizing traits (SS and IMP). The relative endorsement of each motive by personality group is shown in Table 7. Relative to other motives: SS endorsed enhancement motives most frequently (29.69%); IMP endorsed enhancement (24.44%) and coping with anxiety, stress, and trauma (28.89%) motives; and AS endorsed enhancement motives (20.69%), coping with depression (20.69%), and particularly coping with anxiety motives (41.38%). Of note, when examining the specific coded content of the broad coping with anxiety, stress, or trauma motive, IMP endorsed a variety of such motives including coping with anxiety, stress, and trauma, while AS endorsed coping with anxiety symptoms almost exclusively. Finally, HOP endorsed both coping with depression (30.43%) and enhancement (26.09%) motives. However, when examining the qualitative nature of the enhancement motives endorsed in HOP interviews, the context surrounding their enhancement motives seemed to relate to seeking energy, waking up, or escaping anhedonic states. As one participant explained: *“I just did not care about anything, like, good or bad, just I felt good and that was all I cared about I guess.” (B208, female, age 29, HOP)*.

**Table 6:** Motives for substance use excerpts.

Motives	Excerpts
Enhancement	“Uhh, the energy, like, $10 worth would last you 3–4 days and you would stay awake for 16–18 h at a time and, and still perform regularly, like you know, you felt like superman, like literally, like there’s nothing you could not do and you were always wide awake, alert, you were not like slurring, or passing out or anything like that so nobody could look at you and think that you are high on crystal meth.” (A118, male, age 42, HOP)
	“And I know if I take a handful of ‘em I’m gonna get high off them. […] I found out real quick if I took two or three of those I was fucked up. […] I knew, I, I realized right away the more pills I took the better I felt. And it was just game on from the minute they put me on them.” (A105, male, age 48, IMP)
Social	“But others, and we’d uh have a couple of tokes of that, play some music, and sort of a social thing, that’s...” (A103, male, age 61, SS)
	“For example, this morning, I got up, I called a friend. He came by we had breakfast -- a coffee, peanut butter sandwich – and we smoked a joint. Because my friend was there. If he wasn’t there, I would have smoked around two o’clock three o’clock.” (C115, male, age 51, IMP)
Expansion	“Exactly, you, you can hang back watch a show or hang with your friends or, or do something on the computer just and it puts an extra spin on the end of it” (C115, male, age 51, IMP)
Conformity	“But it was also because I wanted to fit in with them there. That’s what they were doing, they smoked. I did not want to be left out, so I started smoking with them, and I made myself some friends.” (C210, female, age 50, SS) [Author translation, original in French]
	“Mmm...TV shows or y’know someone else that’s using beside me. Y’know, like before I had gotten married uh... y’know I was using opiates and people were smoking crack beside me and shootin’ it up and, or shootin’ up their pills and y’know...before I would just say fuck it and I’d- and I’d do up a head or I’d smoke this or smoke that.” (A154, male, age 43, IMP)
Coping with anxiety, stress, or trauma	“I used to rely on Benzodiazepines and alcohol. When I was younger. To... To keep myself calmed down and relax and all that that but…the main reason I smoke marijuana now too … at-you know- three four grams a day … the only reason I’m using so much right now is ‘cause I’m all stressed out.” (A105, male, 48, IMP)
	“Without it sittin’ there torturin’ your brain and your mind too. ‘Cause that’s why people will use, … most people that use drugs and alcohol… betcha 90 %’s been abused. In some form or way. Sexually, physically, mentally, what have you.” (B143, male, age 42, IMP)
Coping with depression	“Thinking about, thinking about different things that have happened in my life, different situations with my family… or if I feel lonely, when I feel lonely or I feel like I do not have anybody that I can turn to, that’s usually when I feel like I need to use the worst” (B136, male, age 28, SS)
	“Numbed out, I guess. Not physically numb, but your body is not numb, but your brain gets fully numb and you just, you do not wanna think. Basically you just wanna sit back and just, enjoy, right? Take it in, I guess. Escape reality, I guess you could call it. It’s an escape.” (C112, male, age 32, HOP)
Coping with withdrawal	“To get that fix for the day an’ then, you go to sleep, the next day you gotta worry with the stress again. The same thing ‘cause you do not wanna be all sick and sore.” (B143, male, age 42, IMP)
	“I do not know, a couple times I could not get my methadone. And I did not wanna be sick all day so I managed to get a pill. And one was just recently. Uhh, last month or somethin’. I could not get my methadone.” (A207, female, AS)
Coping with pain	“It did not work for very long and then somebody introduced that crystal meth and it seemed to take all my pains away, all aches/pains I could- I was a new man after that and then I hid it for years and years and years.” (A118, male, age 42, HOP)
	“I uh, you know I’ll eat uh some morphine pills or whatever... Something to just kinda relax me ‘cause I do not think, like they say methadone is like helps the pain instead of- it helps the pain from the … prior opiate use, it does not help body pain.” (A154, male, age 43, IMP)

SS, sensation-seeking; IMP, impulsivity; AS, anxiety-sensitivity; HOP, hopelessness.

**Table 7:** Motives for substance use endorsement by personality type.

Motives for use	SS (5)	IMP (6)	AS (4)	HOP (4)	Total (19)
Conformity	1.56%	3.33%	3.45%	4.35%	2.91%
Enhancement	29.69%	24.44%	20.69%	26.09%	25.73%
Expansion	1.56%	2.22%	0%	0%	1.46%
Social	9.38%	2.22%	0%	4.35%	4.37%
Coping with pain	6.25%	10%	0%	8.7%	7.28%
Coping with anxiety, stress, or trauma	17.19%	28.89%	41.38%	21.74%	26.21%
Coping with depression	17.19%	18.89%	20.69%	30.43%	19.9%
Coping with withdrawal	17.19%	10%	13.79%	4.35%	12.14%
Total % (# references coded)	100% (*n* = 64)	100% (*n* = 90)	100% (*n* = 29)	100% (*n* = 23)	100% (n = 206)

SS, sensation-seeking; IMP, impulsivity, AS, anxiety-sensitivity; HOP = hopelessness. Number in parentheses indicates the number of interviews conducted with each profile. Cells are color heat mapped to visually indicate the relative endorsement of each theme within each respective personality group. Red indicates high endorsement, yellow/orange is moderate endorsement, and green is low endorsement.

## 4. Discussion

This study employed a mixed methods design combining thematic and content analysis to investigate the lived experience of MMT clients scoring highly on SURPS personality traits. Our two goals were to extend theory on the four-factor personality vulnerability model for substance use (21) among MMT clients, and to provide client-informed material for future personality-targeted manual development for an intervention to reduce polysubstance use in MMT clients. These lived experiences were described through three major themes, which included symptoms of internalizing and externalizing forms of psychopathology, adverse experiences, and polysubstance use.

When asked to tell a story about how their personality got them into trouble, participants described a variety of thoughts, affects, and behaviors that fell under a general theme describing various internalizing (i.e., a tendency to express distress internally, such as depression, anxiety and fear) and externalizing (i.e., a tendency to express distress externally, including substance use problems and behavioral problems) psychopathology symptoms, which correspond to existing and well known quantitatively derived transdiagnostic models of psychiatric comorbidities (i.e., internalizing-externalizing model; see (31, 32)). In this study, MMT clients reported internalizing symptoms comprised of depression, anxiety, panic, and avoidance. They also reported externalizing symptoms of disinhibition (difficulty controlling impulses and not thinking about the consequences of actions), anger, thrill-seeking, aggression, and other anti-social behaviors (e.g., criminality). These symptoms were heavily intertwined with substance use and other adverse experiences, including trauma exposure, poverty, health problems, interpersonal problems, and marginalization/stigmatization. This intertwining highlights the complex interplay between these factors and the need for additional psychosocial services for MMT clients that can address these complex comorbidities (33–35).

### 4.1. Personality targeted model

This study provides additional validation of the four-factor personality vulnerability model (21, 23, 36) in MMT clients by demonstrating personality-specific patterns of internalizing and externalizing symptoms of psychopathology, preference toward use of specific substances, and preference toward specific motives for substance use. Our findings suggest that personality-targeted treatment manuals can be designed to target specific cognitive, affective, and behavioral patterns within each personality type, and our thematic findings highlight which themes are most important to address in each manual. These interventions are typically delivered in a group format, with members of a specific personality profile meeting together. The manuals for personality-targeted treatment typically include scenarios which are composites of stories told by people with lived experience of the traits in question. Although most of the manualized implementation of this model has been done with youth (e.g., the Preventure program, as described in (19)), some of the initial manualized work has focused on adults with substance use disorders and shown promise in reducing frequency and severity of problematic alcohol and drug use (36). Our interview findings provide useful material for inclusion in the creation of such scenarios for future use with MMT clients, that will make the manuals and intervention content more personally relevant to their lives. Our results also highlight that high-risk personality traits in clinical populations are often comorbid, with 73.8% of our sample scoring high on more than one SURPS personality trait. This is significantly higher than reports in non-clinical populations indexing this rate at 36% [e.g., (37)]. This suggests the possibility that individuals in MMT may benefit from skills learning from several different personality-targeted groups.

#### 4.1.1. Sensation-seeking

MMT clients scoring high in SS described a thrill seeking, reward sensitive cognitive style that drives them to take risks and break rules (including engaging in crime). They also endorsed a moderate level of depressive symptoms and traumatic events. The preference toward increasing their arousal levels may predispose individuals high in SS to engage in risky activities with negative consequences (e.g., experiencing trauma, binge drinking and getting injured, chasing a high and risking an overdose), highlighting that treatment manuals with high SS MMT clients should focus on encouraging more safe and effective ways to meet their needs for arousal and excitement (38). Consistent with theory (21), motives for substance use among SS largely focused on enhancement (i.e., to get high, for pleasure), but also included a variety of coping motives, similar to prior research highlighting that clinical populations of persons who use substances engage in coping motivated use (39).

#### 4.1.2. Impulsivity

MMT clients scoring high in IMP described a combination of both externalizing and internalizing symptoms of psychopathology. This group was characterized by themes reflecting a general tendency to act automatically without thinking through the consequences, or a feeling of not having control over one’s actions. This personality profile also reported more instances of anger, frustration, and aggression, relative to the other profiles, and showed a moderate endorsement of depression, anxiety, and stress. These themes were intertwined with a wide range of adverse experiences, including interpersonal conflict, marginalization, health problems, and trauma. High IMP clients also referenced heterogenous motives for use which included both positive reinforcement (e.g., enhancement) and negative reinforcement (e.g., coping with anxiety, stress, or trauma and coping with depression) motives. This personality profile also tended to endorse engagement in various types of substance use, thus showing a pattern of polysubstance use. Treatment for high IMP MMT clients may need to address not only cognitive disinhibition and its’ consequences, but also provide effective emotional regulation strategies, particularly for addressing depression, anxiety, and stress. Interventions to curb polysubstance use in IMP clients will need to recognize their heterogenous substance use motivational profiles.

#### 4.1.3. Hopelessness

By and large, MMT clients scoring high in HOP described an internalizing profile of psychopathology symptoms consisting of high levels of distress, sadness, and depression. Their descriptions frequently included stories of loss, relationship ruptures, low social support, and chronic health problems, including experiencing chronic pain. HOP is a known risk factor for depression (36) and health problems (40, 41). Additionally, depression and depression-like traits (i.e., hopelessness) and chronic pain have bi-directional associations (42, 43). These multiple risk factors likely play an important role in the emergence and/or maintenance of the internalizing symptoms reported by our high HOP group and will need to be carefully considered by clinicians. Additionally, the use of deception to avoid negative interpersonal consequences featured more prominently in descriptions given by high HOP people. This is in line with a recent study in adolescents which showed a unique positive association between HOP and high scores on lying and cheating, even when accounting for links of HOP with general psychopathology and higher order distress (44). Discussions around substance use often focused on opioids as a form of coping with these internalizing symptoms, but also included stimulant use (i.e., crack cocaine) to provide energy and to escape feelings of anhedonia. Treatment for high HOP MMT clients will need to consider not only effective alternative strategies for coping with low mood and interpersonal conflict, but also include alternative strategies for coping with pain or health problems [e.g., see (45)] and for achieving energy and pleasure given their propensity for anhedonia.

#### 4.1.4. Anxiety sensitivity

Clients scoring high on AS reported an internalizing profile largely characterized by anxiety, fear, and avoidance coping strategies. Consistent with prior research linking AS with panic disorder (46), experiences of panic symptoms (e.g., catastrophizing about physical sensations) and fear were frequently discussed by this group. Although all profiles described the unpleasantness of opioid withdrawal symptoms, these descriptions occupied more of the overall discussion among AS individuals. Health problems were also frequently discussed among AS clients, which may be due to their enhanced somatic sensitivity and high levels of health anxiety (47). Although trauma was not discussed at all in the AS interviews, this likely was because the interview guide did not explicitly focus on trauma in combination with high AS individual’s endorsement of avoidance-related coping strategies which may have included avoidance of speaking about trauma. Use of tranquilizer drugs such as benzodiazepines were frequently discussed. These were often prescribed, and largely used for coping with anxiety. Similar to recommendations from other investigators (48, 49), psychosocial treatment for high AS MMT clients will need to consider addressing symptoms of anxiety, panic, and other negative affective states.

### 4.2. Limitations

Our findings have several important limitations. While the addition of content analysis to our thematic analysis is a strength of this study as it allowed to describe thematic findings visually and numerically across groups, it is important to recognize that percentage endorsement of substance use type may not necessarily map with actual frequency of use (i.e., participants may have chosen to talk about certain substances more frequently). Additionally, although our total sample size was sufficient to reach saturation for identifying a variety of deductive and inductive themes (50), it was relatively small at the sub-group level, and some personality groups (e.g., IMP) were more talkative than others (e.g., AS), thus providing more data. Although we focused on within-personality coding to attenuate the influences of this discrepancy, it is possible that additional themes may have emerged from recruiting more participants, particularly AS participants. Additionally, AS as measured by the SURPS is largely limited to measuring physical concerns relating to anxiety symptoms, and does not measure cognitive and social concerns. Although our AS participants reported mental and social concerns in their interviews, future studies may consider using other well-validated but longer measures of AS (e.g., the Anxiety-Sensitivity Index-3, (51)) which measure this personality trait more broadly. Further, our OAT sample exclusively included people on MMT, and may not generalize to other forms of OAT such as buprenorphine/naloxone. Buprenorphine/naloxone has an improved safety profile, and thus permits more flexible at-home dosing schedules, but may be less efficacious for those who are more severely opioid dependent (52–54). Another limitation of this work is that the interviewers were not blind to the personality of the interviewee and interviewees were specifically told they were interviewed because of scoring high on one of four specified personality traits. Although this was necessary here as the goal was to collect specific information for the development of manual content, future qualitative studies may consider employing interviewers blind to personality status and asking non-specific questions about the links between personality and behavior to eliminate the risk of potentially limiting the range of responses given by interviewees. Additionally, most of the participants were middle-aged and White. While this is representative of the demographics of those at the clinics where recruitment took place, it is possible that additional or different themes might emerge among younger or more diverse OAT clients. Finally, participation in the study was voluntary; these themes may not generalize to OAT clients who would not want to engage in semi-structured interviews.

### 4.3. Conclusion

This study provides additional qualitative support that personality remains an important correlate of various cognitive, affective, and behavioral processes among MMT clients including polysubstance use (24). Themes identified through this study can be adapted into “scenarios” for future adaptations of existing personality-targeted treatments (19) to reduce distress, externalizing behaviors, and polysubstance use among MMT clients. Future studies should consider examining the various symptom clusters, motives for use, and adverse experiences identified in these interviews and their relationships with personality using quantitative methodologies among clients struggling with opioid addiction.

## Data availability statement

The original contributions presented in the study are included in the article/Supplementary material, further inquiries can be directed to the corresponding author.

## Ethics statement

The studies involving human participants were reviewed and approved by Nova Scotia Health Authority (Nova-Scotia) and Le Comité d’éthique de la recherche (CÉR) du CHUM (Quebec). The patients/participants provided their written informed consent to participate in this study.

## Author contributions

Under the supervision of SS, IM developed the research questions and hypotheses, completed a portion of the data collection, prepared the dataset for analysis, conducted the analyses, and interpreted the study findings. SS was the principal investigator for the Nova Scotia research team, while PC was the principal investigator for the Quebec research team, which both participated in the recruitment and data collection efforts. AS and JS both helped with recruitment and data collection. JS further assisted with transcription of interviews. SS, SB, and PC supervised the development of the interview guide. All authors reviewed the manuscript and made intellectual contributions.

## Funding

Data collection was possible through funding from the Canadian Institute of Health Research (CIHR) CRISD grant 139149 which was part of a demonstration project funded by the Canadian Research Initiative in Substance Misuse (CRISM).

## Conflict of interest

The authors declare that the research was conducted in the absence of any commercial or financial relationships that could be construed as a potential conflict of interest.

## Publisher’s note

All claims expressed in this article are solely those of the authors and do not necessarily represent those of their affiliated organizations, or those of the publisher, the editors and the reviewers. Any product that may be evaluated in this article, or claim that may be made by its manufacturer, is not guaranteed or endorsed by the publisher.

## Footnotes

^1^Due to an administrative error at one site, an AS participant completed an IMP interview – but given that this participant also met criteria for high IMP, their data was retained for analysis.
