# Supplementary material for: The four-factor personality model and its qualitative correlates among opioid agonist therapy clients
Source: Front Psychiatry. 2023 Jun 9;14:1129274. doi: 10.3389/fpsyt.2023.1129274 (PMC10289030; doi:10.3389/fpsyt.2023.1129274)
Supplement: Supplementary file 1 [file Table_1.DOCX]

**Appendix : Semi-Structured Interview Guide**

**[note: the entire interview, including this explanation will be read orally by interviewers]**

This is an interview about your personality, your substance use, the settings in which you use drugs, your triggers for drug use and your reasons for drug use. **We are interested in hearing stories about your typical or general drug use habits, but also some specific recent situations when you used drugs, to clarify your moods and reasons for using drugs**. We are particularly interested in hearing about how your personality may sometimes get you into trouble. Following some of the questionnaires you completed, we noticed that you indicated that you *[Read out relevant items from the SURPS; e.g. “That you would really like skydiving” or “that unusual physical sensations scare you” ]*. Our goal is to understand how coping with these personality traits influences your drug use behavior.

The interview should not be seen as a “therapy session.” This interview is for research purposes only, and its sole purpose is the collection of data concerning people’s drug use while receiving methadone maintenance treatment to adapt an existing intervention to improve coping skills that is designed for people like you. At the end of the interview we can give you resources on mental health services that you can access at your own discretion. Your responses are fully confidential: However, please keep in mind that we may have to share your information with the proper authorities if you talk about abuse or neglect of a child, an adult in need of protection, plans to commit suicide or planning to harm another person. Other than those exceptions, all of your responses, including you reporting any substance use, will be kept confidential.

This interview will be audiotaped. All information you shared today will be de-identified to protect your confidentiality. This means we will remove any and all identifying information (e.g., names, place names, street names). At the end of the interview you will have the chance to decide whether you want your audio recording to be used for our research purposes or to be deleted.

Initially we will talk a bit about your personality traits and how they may have gotten you in trouble in the past. From there we will move into more specific questions about your experiences with drugs and how you think your personality style shaped those experiences. We are looking for details about what happened before you started using drugs, what happened while you were using drugs, and what happened after, including what you were thinking, feeling, and doing at that time. Please feel free to share as much or as little as you are comfortable with, and to ask me any questions you have as we go along.

Before we begin, do you have any questions? [~2:20 minutes]

***Note: at this point, the interviewer will start audio recording the interview and inform the participant of this.***

**Section 1. Goals & Priorities**

**Guidelines:** We want to know what their priorities, needs & goals are.

**Thank you for talking with us today. Before we begin with some of our other questions, we would like to get to know you a little better. Tell us a bit about what your goals and priorities are. Where do you see yourself in the near future?** (if only short-term goals offered, prompt for long-term goals *if they have any*)

____________________________________________________________________________________________________________________________________________________________

**In respect to your methadone treatment, what are your goals** (e.g. do you want to stop using completely, stop using certain substances, or keep things as they are)**?**

____________________________________________________________________________________________________________________________________________________________

**What is the most important thing in your life at the moment? What are your priorities now? What are your needs?**

____________________________________________________________________________________________________________________________________________________________

______________________________________________________________________________

**What are some of the major barriers in your life?**

____________________________________________________________________________________________________________________________________________________________

______________________________________________________________________________

**Goal Setting & Insight Prompts**

*General: In general, want to get a sense of their motivations. Start on a positive note and see how they frame their goals.*

1. Can you tell me about your goals? What would you like to accomplish or what would you like to change?
2. In terms of your life as a whole, what do you see as the most negative effects or consequences of your drug use? Do you see any positive effects of your drug use?
3. Why is it important for you to receive methadone maintenance therapy?

**Section 2. Personality Psychoeducation & Rapport**

**We are talking to you today because*…****[Pinpoint the relevant items on the SURPS subscale that were highly rated, and re-read them to the participant as a reminder]*.

*Introversion/Hopelessness Dimension (H) Anxiety Sensitivity Dimension (AS)*

Item 1 Item 8

Item 4 Item 10

Item 7 Item 14

Item 13 Item 18

Item 17 Item 21

Item 20

Item 23

*Impulsivity Dimension (IMP) Sensation Seeking Dimension (SS)*

Item 2 Item 3

Item 5 Item 6

Item 11 Item 9

Item 15 Item 12

Item 22 Item 16

Item 19

Use their language whenever possible. Associated emotional vocabulary for use in future prompts:

- H: sad, stressed, depressed, pessimistic
- AS: nervous, tense, anxious, stressed out, overwhelmed, on the edge, unpleasant bodily sensations
- IMP: stressed, angry, frustrated, excited
- SS: bored, “party-time”, “fun”, excited

**Do you recognize yourself in some of these statements? They represent a personality trait known as** *[Negative Thinking/Anxiety-Sensitivity/Sensation-Seeking/Impulsivity]*. **Everyone has some** *[name trait]* **inside of them; some people have more, some have less. For example, I also identify with** *[pick one item from target subscale that is personally relevant]*.

**Sometimes, these personality traits make us act in certain ways, both good and bad. For instance, people**

- [**H**] With negative thinking tend to see the world more realistically, but may sometimes be more vulnerable to bad events and see things as more negative than they actually are.
- [**AS**] With Anxiety-sensitivity are generally more careful, but it may sometimes make them feel more uncomfortable, nervous and tense in certain situations.
- [**IMP**] Who are impulsive have an easier time making decisions and going for what they want, but this may lead them to sometimes get angry easier, have things spiral out of control or make poor decisions because they sometimes act without thinking.
- [**SS**] Who are sensation-seekers typically like to live life on the edge, be social and explore, but this can sometimes get them into trouble through doing dangerous activities.

**How do you identify with this? Does it describe you well as a person?**

**What is** [*negative thinking/anxiety-sensitivity/impulsivity/sensation-seeking*] **like for you?**

____________________________________________________________________________________________________________________________________________________________

**What kind of things does** [trait] **lead you to do?**

____________________________________________________________________________________________________________________________________________________________

**What kind of things do you do to cope with feeling** [AS: stressed out/anxious/nervous/tense; NT: sad/feeling down; SS: bored/on the edge; IMP: angry/on the edge]?

____________________________________________________________________________________________________________________________________________________________

[*words*] **How would you describe** [*trait*]**? What words would you use?**

____________________________________________________________________________________________________________________________________________________________

**That’s very interesting, thank you for sharing. Let’s talk a bit more about how** [trait] **may make you act in certain ways**.

**Section 3. The Situation – Personality & Substance Use**

**General***: You want to obtain information on the situation, the experienced physical sensations, the associated thoughts, and the resulting behavior. Use prompts below as needed.* ***This is the most important page. Use more than one page if necessary or if several situations are described.*** *By the end, it should be very clear why they reacted that way; if you still don’t understand, you haven’t explored the issue enough.*

**[Situation] Can you tell me more about a situation in which these thoughts/feelings/traits got you into trouble?** *[Look for details about:* ***Situation*** *(who, where, when),* **Physical Sensations, *Triggers, Thoughts*** *(self-talk, cognitive distortions, opinions),* ***Behaviors*** *(drug use, risky),* ***Type of substance use****,* ***Consequences****]*

- **What is the context of the drug using event**? E.g., how do they get drugs, where and when do they use them, who facilitates use?
- **If they don’t volunteer a drug use situation, then specifically ask them about illicit drug use.** E.g. “Your [trait] led you to use?”, “Tell me more about a situation in which [trait] led you to use”.

______________________________________________________________________________________________________________________________________________________________________________________________________________________________________________________________________________________________________________________________________________________________________________________________________

**[Physical Sensations] What did you feel in your body in that situation?**

____________________________________________________________________________________________________________________________________________________________

______________________________________________________________________________

**[Thoughts] What kind of thoughts did you have in this situation?**

__________________________________________________________________________________________________________________________________________________________________________________________________________________________________________

**[Action] What did you end up doing? How did you react?**

________________________________________________________________________________________________________________________________________________________________________________________________________________________________________________________________________________________________________________________

**Situation Prompts**

*General: Need detail about the physical situation but also the larger context and triggers.*

1. Where were you at that time?
2. What time of day was it? E.g. do they use in the morning, afternoon or evening?
3. What season?
4. Why were you there? What were you supposed to be doing at the time?
5. How did you get drugs? Who facilitates this?
6. Where did you use?
7. Who else was there?

**Physical Sensation Prompts**

*General: How do they experience the personality trait? Examples include feeling rapid heartbeat, muscle tension, difficult breathing, upset stomach, sweating, trembling, headaches, shaky voice, butterflies in stomach, sweaty palms, agitated, dizzy, feeling sick.*

1. What did you feel in that situation?
2. How did it feel inside your body?
3. Do you remember feeling a particular part of your body react?
4. Was your heart racing?
5. Did you notice your hands or shoulders tensing?
6. Did you feel restless?

**Personality & Thoughts Prompts**

*General: pay attention to how personality traits in specific situations seem to trigger substance use and risky behavior.*

1. What kinds of thoughts did you have in this situation?
2. [**IMP, AS**] When you experience conflict or frustration, what do you do? [**IMP**; *Risk*] Do you sometimes do dangerous things or things that other people would think are dangerous?
3. [**H, AS**] When you experience stress, what do you do?
4. [**H**] When you experience a sad, low, or depressed mood, what do you do?
5. [**AS**] When you feel nervous, tense, or anxious, what do you do?
6. [**SS**] When you feel bored, what do you do? [*Risk*] Do you sometimes do dangerous things or things that other people would think are dangerous?
7. [*Coping*] Is there anything that you can think of that makes you better cope with [these feelings, thoughts, personality]?
8. [*Thoughts*] Are you aware of any inner thoughts or emotional feelings, or things within you as a person, which trigger off your need or desire to take the drug at a particular moment in time?
9. [*Risk*] Would there be any specific feelings or situations that might be more difficult for you to handle?
10. [*Protective*] Can you describe a situation or set of events which would be least likely to result in your feeling like using drugs? In other words, when do you feel least inclined to use drugs?
11. [*Withdrawal*] Suppose that we were to decide that you would not use drugs at all for the next two weeks. What problems do you think you might have if you did this? How do you think it would affect you?

**Action/Behaviour Prompts**

General**:** The result of the thought on behavior in that situation.

1. What did you end up doing?
2. How did you react?
3. Is this the reaction you wanted to have?

**Substance Related Prompts**

*General: pay attention to how drugs & their effects are described, polysubstance use, drugs as coping mechanisms.*

(*prompt if necessary*): **I see that you’ve reported using** [*name the drugs that the participant reported using in their substance use questionnaire*]. **I would like to know more about how you feel towards** [*drug*(s)], **what you like about them, if you have any concerns surrounding them, and the way in which you use** [*drug*].

1. When do you typically use, where, how many times in a day will you use?
2. Which drugs do you think you are most at risk using while on MMT
3. What do you like best about the drug?
4. What do you think influences your drug use? What makes it difficult to stop or to resist?
5. [*Motives*] What are the main reasons why you use drugs? In other words, when you are actually using drugs, what for you is the most positive or desirable effect of the drug for you?
6. When you use *(pick depending on what participant endorses previously)*
   1. to reduce [AS: *anxiety/*IMP*: stress or boredom/*SS*: boredom/*H*: sadness*]
   2. or to [AS: relax/ IMP: cool down, relax or get the party going/ SS: get the party going, H: feel better], what do you do? What happens?
7. So it makes you feel less [*anxious/impulsive/bored/sad*], what do you do next?
8. How does that work for you? Then what do you do?

**Self-Efficacy/Treatment response prompts**

1. Do you think this is something you can change?
2. [*Concerns*] Can you tell me more about any worries you might have about your drug use?
3. Any workshops available at your clinic already? Are they helpful?

**Finish with a brief summary/reflection of the situation at the end to see if the participant offers any corrections.**

**Thanks so much for your time and for sharing your story with me, I really appreciate it. Is there anything you would like to add?**

[Linking with manual] **I can understand how you would act that way if you go through this situation. Hopefully, our treatment will be able to address some of these issues and we are hoping to use stories such as yours as examples in our manual.**

Space for additional notes:

____________________________________________________________________________________________________________________________________________________________________________________________________________________________________________________________________________________________________________________________________________________________________________________________________________________________________________________________________________________

**Section 4. Manuals & Intervention**

*General: Probe how they feel this intervention fitting in their routine and their program/clinic.*

**Now I have some general questions about the format of a manual we use in a personality treatment with adolescents and adults and I would like to have your opinion on how you think this treatment could be delivered to adults in methadone maintenance therapy. I’ll show you some examples of manuals and pamphlets, feel free to add these post-it notes to sections that you find appealing or interesting or that you think we should change.**

**Manual Prompts**

1. How do you feel about using a booklet?
2. If you were participating in this treatment, would you like to take the manual home in between sessions?
3. Is there too much text?
4. If we were to change the illustrations, what would you like to see?
5. Are you open to completing exercises on “thoughts” in the manual?

**Intervention Prompts**

1. *[Intervention interest]* How interested would you be in additional services at the clinic, centering on building better coping skills for managing [*stress, boredom, anxiety, sadness*]? Why would it be important for you to have these services, if you are interested?
2. [Trauma] We recognize that trauma plays an important role in addiction. But there’s a time and place in addressing it in recovery. This intervention provides coping skills that help with coping with trauma, but does not address it directly. How do you feel about this approach?
   - *Not necessarily interested in the content, but rather the impact trauma has on their arousal and cognition. What we would not do is ask them about what happened. Rather explain how trauma may be addressed and ask them about it.*
3. [*Confidentiality*] We recognize that reporting substance use may affect your treatment. How confident are you to discuss it with us? Is there someone at your clinic that you feel can help you with this?
4. [*Confidentiality*] In this format, with me, did you feel truthful? If you were to be honest with me, how comfortable did you feel telling me the truth (%)?
5. [*Confidentiality*] Would you take it up if we provide you with the same confidentiality in an actual treatment?
6. How well do you read when you are on methadone or any other psychiatric medication you may be taking?
7. How would you like to see the session delivered (i.e., groups of similar people or individual format)?
8. Based on your experience at the clinic, do you think a group session is practical (i.e. doable)?
9. If the intervention sessions would be delivered in a group format, would you be open to sharing your story or experiences with other clients with a similar personality style from your clinic?
10. How long do you think a session should be? How long is too long before you lose interest?
11. Would you want the intervention sessions to be delivered by the ‘experts’ (people who developed it) or by the clinic staff whom the clients already know?

**Thanks so much for sharing your opinions on these manuals with me, I really appreciate it. Is there anything you would like to add?**

**This session has been audio taped. All information you shared today will be de-identified to provide anonymity and protect your confidentiality. This means we will remove any and all identifying information (e.g., names, place names, street names). Are you still okay if we keep the audiotape and use this information in our research for the purposes of helping us to modify and develop the intervention manuals?**

Proceed to compensation.

**Appendix : Semi-Structured Focus Group Guide**

**The purpose of this focus group is to get your opinions on how we can adapt an intervention for youth and adults, for the methadone maintenance population. Let’s take a look at the manual that currently exists for youth and the pamphlets we have for adults, and get some feedback on how we can change it for the methadone maintenance population. The goal is to be able to adapt this intervention so we can use it with the population and once it is developed, another study will pilot how well it works with MMT clients.**

1. Is there too much text?
2. Are the illustrations relevant or do they need to be changed? How would they best be changed?
3. Is there anything in the text you feel is missing and that you feel would be helpful?
4. What is the local terminology for different drugs? What do your clients call them?
5. Would the interview using the new intervention work best in a group format or an individual format?
   1. What is more practical within your clinic? Can a sub-group of clients all meet at the same time?
   2. Will your clients do better in a group or individual format? What client characteristics would influence this?
6. How many sessions is an ideal length for an intervention of this sort (i.e. how many sessions would a client at this clinic be willing to commit to?)
7. What is the ideal length for such an intervention within this population? How long is too long before they lose interest?
8. What client characteristics, if any, would determine who would be most interested/motivated in this intervention?
9. Would clients want this intervention delivered by the ‘experts’ (people who developed it) or by the clinic staff whom the clients already know? What is your preference at the clinic?
10. Any other advice on the intervention that may be relevant for developers?
